# The Influences of Brand Awareness on Consumers’ Cognitive Process: An Event-Related Potentials Study

**DOI:** 10.3389/fnins.2020.00549

**Published:** 2020-06-12

**Authors:** Xuefeng Zhang

**Affiliations:** School of Management, Southwest University of Political Science and Law, Chongqing, China

**Keywords:** brand awareness, identification, attention, event-related potentials, N2, P3

## Abstract

Brand awareness plays an important role in most aspects of marketing. However, consumers’ cognitive process of brand awareness, which plays an important role in purchase decision or product usage experiences, is still unclear in the brain. Using event-related potentials (ERPs), the influences of two different brand awareness on consumers’ cognitive process was investigated. Phone pictures with high or low brand awareness and girl pictures were used to carry out this experiment research. An amended oddball task was designed in which girl photos were taken as target stimuli, and phone pictures were taken as non-target stimuli. Subjects were asked to identify the girl pictures. Smaller ERPs components N2 and P3 along with high brand awareness phone pictures were found compared to the low brand awareness ones. The amplitude variation in N2 and P3 indicated that the cognitive process of identification and attention distribution were changed along with the magnitude of brand awareness, which meant consumers could allocate different attention resources to distinguish high or low brand awareness product unconsciously. This may indicate the identification and attention distribution caused by brand awareness can be detected by N2 and P3, and event-related potentials methodology may be a sensitive measurement technique for brand awareness.

## Introduction

As is known to all, brand awareness plays an important role in consumer decision-making, market performance, marketing mix, and brand equity. Keller (2008) have pointed out that brand awareness refers to whether consumers can recall or recognize a brand, or simply whether consumers know about a brand. Just as people buy mobile phones, people are more inclined to buy iPhone or Samsung than a less well-known brand, such as Smartsan or UooGou. Obviously, when consumers face a vast commodity brand, the higher the brand awareness, the easier it is to attract consumers. Scholars have conducted extensive research on the two important aspects of brand awareness, brand recognition and recall. Most scholars have reached a consensus that brands recognition and recall are important while consumers are making purchase decisions (Emma and Sharp, 2000; Thoma and Williams, 2013) and evaluating product usage experience (Huang and Sarigoellue, 2012; Stach, 2019) or product quality (Huang and Sarigoellue, 2012). Brand recognition and recall also affect consumer attitudes and emotion (Rossiter, 2014; Wilson et al., 2015; Xu et al., 2015), even the firm performance (Grundey and Bakowska, 2008; Homburg et al., 2010).

However, most of the research mentioned above mainly adopted questionnaire (Emma and Sharp, 2000; Koenigstorfer and Groeppel-Klein, 2012; Topolinski et al., 2014), interview (MacInnis et al., 1999; Turner et al., 2015), or market survey (Naik et al., 2008; Homburg et al., 2010) as research methods. Despite growing concerns about cognitive research on brand recognition and recall from the perspective of psychology, such as studies on purchase intention (Ashby et al., 2015; Topolinski et al., 2015), memory (Valkenburg and Buijzen, 2005; Hubert et al., 2013), or categorization (Esch et al., 2009; Dew and Kwon, 2010), etc., research into the cognitive process of brand awareness in the brain has not aroused sufficient attention. Even though the approaches above are easy and cheap to implement, the data gathered may include biases. As a consequence, the results of those studies usually do not match the actual behavior of consumers when they buy (Scheier, 2007). The reason for the deviation lies in the unavoidable shortcomings of the above-mentioned methods itself. Whether it is for the collective, the individual, and whether it is conducted in a confidential manner, researchers still rely on consumers’ self-reports to investigate their response to brand awareness. But these methods have limitations. First, the researchers assumed that the respondents were able to describe their own cognitive processes, but in fact, many of the subconscious parts of the process were not known to the respondents. Second, there are many other factors, including incentives, time constraints, or peer pressures, that induce respondents to distort their feelings, so that the results of the survey do not fully reflect the true thinking of the respondents. It is important to note that some earlier studies have already involved the cognitive process and brand awareness in the brain, such as linguistic encoding and retrieval processes of brand experiences (Esch et al., 2012), the basis of the relationship between brand personality associations and brain activity (Chen et al., 2015), the consumer-psychology model specification synthesized psychological constructs and empirical finding using consumer-neuroscience methods (Schmitt, 2012). However, studies on the neural response to brand awareness straightforward have not yet started, which can provide marketers with a theoretical foundation. Therefore, marketers and researchers need to re-examine research methods in order to better understand consumer behavior (Pozharliev et al., 2017).

By using neuroimaging tools, consumer neuroscience might be cheaper and faster than current marketing tools and better understand the decision-making and related processes than usual (Ariely and Berns, 2010; Plassmann et al., 2015). With these advantages, consumer neuroscience has attracted scholars’ interest and attention since the “Coke and Pepsi” experiment (Hoegg et al., 2010). People realize that by observing the processing of stimuli in the brain, we can study how consumers respond to external stimuli and to observe and study consumer behavior from the neuroscience level (Scheier, 2007). Based on the theory of consumer neuroscience and earlier event-related brain potential (ERPs) studies (Sutton et al., 1965; Roth, 1973), we expect brand awareness to be intrinsically related to brain neural response. In this study, we take phones with different brand awareness, the ordinary wireless communication devices, as research objects to carry out the cognitive research by ERPs. We hypothesize that the process of consumers’ brand recognition and recall will be activated and reflected spontaneously by ERPs components alone with different brand awareness, which might be sensitively associate with the cognitive process such as categorization and memory retrieval according to the literature (Polich, 2007; Folstein and Van Petten, 2008). The study could provide a novel way to estimate brand awareness from another perspective, especially when traditional evaluation methods are controversial, the evoked components may serve as a sensitive indicator.

## Materials and Methods

### Subjects

Sixteen right-handed college students (eight male and eight female; mean age 25.6 ± 2.8 years) were recruited. All subjects reported normal or corrected-to-normal vision. All of them were free of neurological or psychiatric illness, head trauma, or drug abuse. Written informed consent was obtained from each subject according to the local medical ethics committee. Subjects received a small gift as compensation after the experiment.

### Stimulation

The critical stimuli were beautiful girl photos, two categories of colorful phone photos. According to the Chinese mobile phone brand reputation report 2018, two phone brands with different brand awareness were chosen in this study. The high brand awareness index was 5.46, and the low one was 2.71. Subjects reported that they had heard of the phone brands before the experiment.

### Experimental Design

**FIGURE 1:**
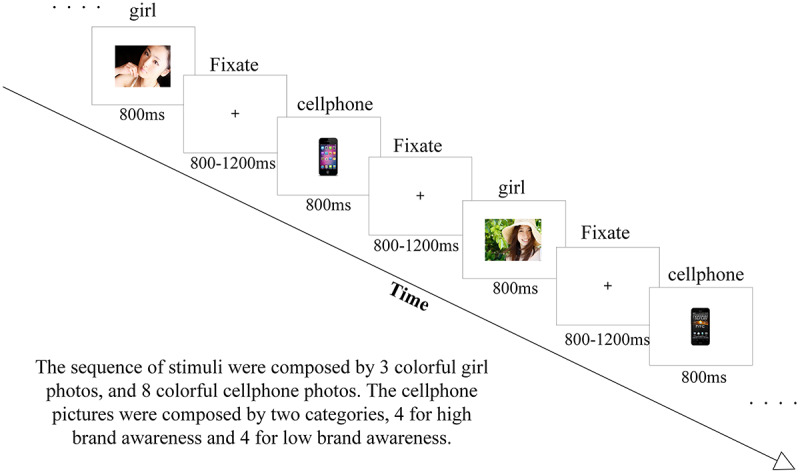
The sequence of stimuli with beautiful girl photos and colorful phone photos in an amended oddball paradigm.

An illustration of the basic classes of stimuli and the timing of the experiment is shown in Figure 1. Subjects viewed a sequence of colorful beautiful girl photos (*n* = 3) and two categories of colorful phone photos (*n* = 4) for each brand. The reason we chose beautiful girl photos is that we want to attract subjects’ attention so that when subjects see these two categories of colorful phone photos unconsciously, we can explore their brain activity. An amended oddball task had been used: the girl photos were taken as target stimulus and phone pictures were taken as non-target stimulus. The target proportion of series was 27.3%.

An electrically shielded and sound-attenuated experimental chamber was used. The Subjects seated in a comfortable chair during the experiment. Each trial began with a screen-centered fixation cross presented in light gray against a black LCD computer screen. The task was programmed and presented by E-prime professional (vision2.0, Psychology Software Tools). Each experimental block contained 11 photos, 3 target stimuli, and 8 non-target stimuli. Each trial presented more than 10 times. One trial consisted of the presentation of the stimulus (duration of 800 ms) followed by a fixation cross (random inter-trial interval with a duration between 800 and 1200 ms) to avoid repeated presentations of the stimuli. Those stimuli were viewed from a distance of 100 cm at the center, visual angle 10.3°, horizontal vertical angle 6.8°. The subject’s task was to identify and verbally report to the researcher the number of target stimuli. If the accuracy was less than 95%, the data would be discarded. Before the experimental blocks, subjects performed one training block to familiarize with the task. Subjects were offered a rest break half-way through the presentation of the stimuli.

### Electroencephalogram Recording and Analysis

Subjects wore a 32 channel electroencephalogram (EEG) cap (Quickcap) during their session, with electrodes placed using the International 10/20 system to record and then estimate the ERPs. The electrodes were referenced to the left mastoid. All electrodes’ impedance was kept below 5 kΩ. Vertical eye movements were monitored with electrodes placed directly below the left eye, and horizontal eye movements were monitored with electrodes placed on the outer canthi of the right eye. Electrode recordings were collected by Nuamps amplifiers (Neurosoft Labs Inc.) with a band-pass of 0.01–100 Hz, a sample rate of 1000 Hz. Offline data was processed using Curry7.0 SBA (Neurosoft Labs Inc.). The dataset with more than 6% of the trials rejected was excluded from further analysis. ERPs were segmented into time locked epoches using the picture onset as a reference. The length of the time window was 1000 ms from 200 ms before picture onset to 800 ms after it (baseline = 200 ms). The averages per channel were low-pass filtered through 50 Hz (24 dB/octave) and were computed on the basis of the EEG elicited in response to brand with different awareness index using within-subject repeated-measures analysis of variance (ANOVA).

## Results

**FIGURE 2:**
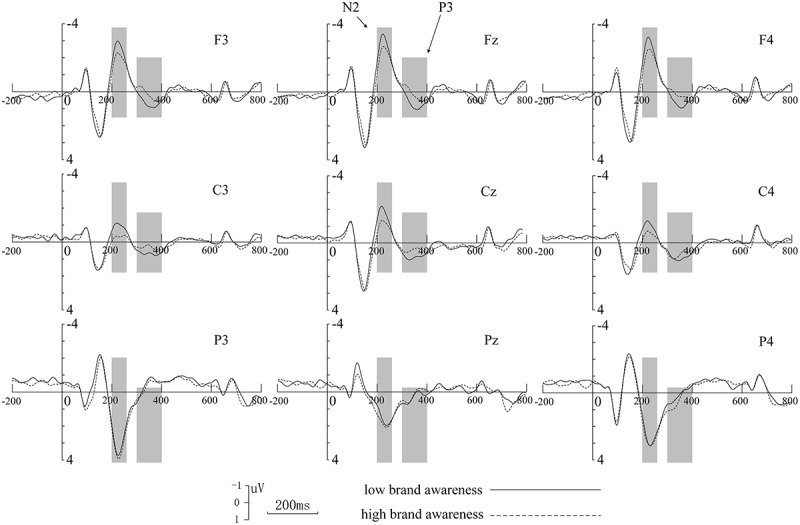
Raw ERPs waveforms at 9 electrode sites. Grand averaged ERPS elicited by stimuli with low brand awareness (solid line) vs. high brand awareness (dotted line) at 9 electrodes in the frontal, central, and parietal areas.

**FIGURE 3:**
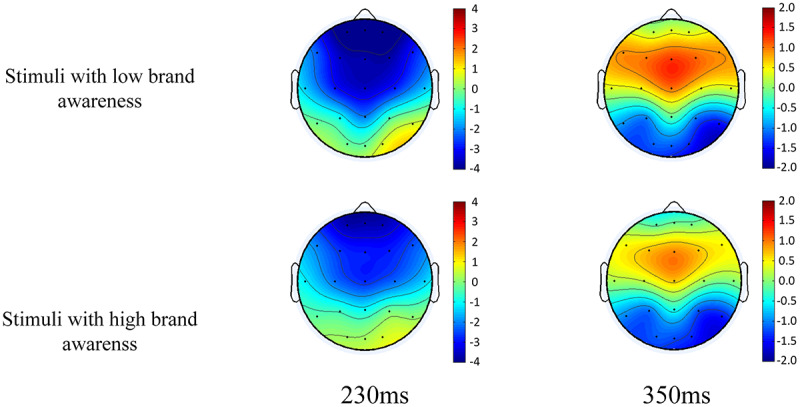
Topographic maps of the voltage field topography. Topographic maps of the voltage field topography at the peak of the N2 and P3 evoked by stimuli with low and high brand awareness. Red and yellow are positive, blue and black are negative, scaled from −4 to 4 mV (N2) or −2 to 2 mV (P3).

The time windows of the ERPs components of interest in the frontal, central, and parietal electrodes were presented in Figure 2. Based on a visual examination of the potential distributions, the scalp topographical mapping of potentials (see Figure 3) and the literature (Potts et al., 1996; Polich and Criado, 2006; Legrain et al., 2009), nine electrodes F3, Fz, F4, C3, Cz, C4, P3, Pz, P4 were chosen for statistical analysis. The average amplitude of N2 in 200–260 ms time window, P3 in 300–400 ms were analyzed. Within each time window, a within-subjects repeated measure ANOVA was used to compare the ERPs (average amplitude of N2 and P3), with brand awareness (high vs. low) and distribution (frontal, central, and parietal) as two within-subject factors. When appropriate, Greenhouse-Geisser correction of degrees of freedom and contrast analysis were used. The significance level was set at *P* < 0.05.

The results showed that, for the N2, there was a significant main effect for distribution [*F*(2, 30) = 29.137, *p* < 0.001], brand variance [*F*(1, 15) = 14.629, *p* = 0.002], and interaction between brand awareness and distribution [*F*(2, 30) = 4.493, *p* = 0.020]. Combining raw waveforms with variance analysis, our study demonstrated that the low brand awareness conditions were associated with higher N2 amplitude than the high brand awareness condition in the frontal and central region, and the highest amplitude of N2 appeared on the Fz electrode when the low brand awareness appeared. Besides, the closer to the front of the scalp, a greater amplitude of the N2 waveform could be seen, whether the stimuli were presented with a low or high degree of brand awareness. For the P3, we obtained significant main effects for brand awareness [*F*(1, 15) = 11.731, *p* = 0.004], but distribution [*F*(2, 30) = 1.592, *p* = 0.220] and interaction between brand awareness and distribution [*F*(2, 30) = 2.778, *p* = 0.078]. Similar to the N2 component, our study demonstrated that the low brand awareness conditions were associated with higher P3 amplitude than the high brand awareness condition in the frontal and central region, and the highest amplitude of P3 appeared in the middle of Fz-Cz electrode when the low brand awareness appeared. Besides, the closer to the front-central of the scalp, the greater amplitude of the P3 waveform could be seen, whether the stimuli were presented with a low or high degree of brand awareness.

## Discussion

The present study aimed at contributing to explore consumers’ cognitive process of different brand awareness through two phone brands. In order to better simulate the impact of brand awareness on consumer cognitive process, we did not try to fabricate a non-existent brand to conduct research. The two phone brands which had different brand awareness on market were chosen to carry out ERPs experiments through a pseudo oddball paradigm. The ERPs results showed significant differences between the two categories of colorful phone photos with different brand awareness. Low brand awareness stimulus elicited higher amplitudes of the N2 and P3 than high ones.

As the result showed, the low brand awareness conditions were associated with higher N2 amplitude than the high brand awareness condition in the frontal and central region. It was generally believed that N2 distributed over the front-central of scalp reflected the process of cognitive control (Folstein and Van Petten, 2008; Liu et al., 2013). We believe that the N2 component could reflect consumers’ identification of brand which corresponded to the recognition process of brand awareness on the basis of previous studies (Schweinberger et al., 2002; Folstein and Van Petten, 2008; Wiese et al., 2014). Stimulation with brands in this experiment had been divided into two categories, one for high brand awareness and another for low brand awareness. According to raw waveforms and statistical analysis, these two categories of phone photos could lead to significant difference in N2 component, we believed that subjects could accurately distinguish the phones’ brand even the phone pictures with a brand awareness difference is taken as a non-target stimulus. As brand awareness was different, subjects allocated more cognitive resources for the stimulus with low brand awareness. Conversely, when stimuli with high brand awareness presented, subject only needed to put less effort into the identification process, therefore the average amplitude of N2 appeared smaller compared to low brand awareness ones. Combining the raw waveforms with topographic maps, we found that the component N2 was mainly distributed over most brain areas from frontal to central area, and its amplitude was lager in Fz than Cz. The result was coincident with previous study (Ernst et al., 2013; Hu et al., 2013).

Similar to the N2 component, the result showed that the low brand awareness conditions elicited higher P3 amplitude than high brand awareness condition, and the P3 mainly distributed over fronto-central scalp. The evoked P3 component in this study might be a P3a-like potential due to its distribution. In general interpretation, the P3, which distributed over the central area, was thought to reflect the allocation of attention (Polich, 2007). The amplitude was observed to decrease as the difficulty of the primary task increased, and thus reflected the attention resources devoted to task performance (García-Larrea and Cézanne-Bert, 1998; Polich, 2007). As mentioned before, the girl pictures were taken as target stimulus, phone pictures were taken as non-target stimulus, and subject’s task was to identify and report to the researcher the number of target stimuli. According to this interpretation, when subjects concentrated on performing the main tasks in the experiment, the phone pictures could be seen as distractions. The higher the brand awareness, the easier it is to be excluded from the task by subjects. As mentioned earlier, the two selected phones’ brand awareness was 5.46 and 2.71, respectively, so that the decrease in brand awareness indexes between brands increased the task difficulty (Hagen et al., 2006) and consequently led to a more intense P3. Another interpretation is that P3a may be subtended by neural changes in the anterior cingulate function when new stimuli replace the contents of working memory (Polich and Comerchero, 2003; Polich, 2007). Compared to the stimuli that had high brand awareness index, subjects were more risk-avoidant considering committing errors and subsequently more likely to activate an anterior cingulate/medial prefrontal network while processing with low brand awareness ones. According to this, the stimuli that had a lower brand awareness could be regarded as a type of non-target distractor. Thus, a larger amplitude of P3 was elicited when the phone photos with low brand awareness presented.

This study had some differences with traditional studies. First of all, unlike the traditional marketing methods such as questionnaire, interview, or market survey, consumers’ cognitive process caused by brand awareness had been studied through an experimental approach. In this study, the relationship between cognitive process and brand awareness was investigated using event-related potentials, and consumers’ cognitive differences caused by high and low brand awareness were explored unconsciously. Second, through the observation of consumers’ cognitive reactions to phone pictures with brand awareness variances, in particular the process of identification and attention distribution, we found that the amplitude deviation of EPR components N2 and P3 could be developed into a cognitive index to measure the process of consumers’ brand recognition and recall, and then to measure brand awareness from another angle.

## Conclusion

To summarize, this study explored the influences of brand awareness on consumers’ cognitive process unconsciously. The result showed that stimuli with low brand awareness could elicit higher N2 and P3 than high brand awareness ones, which means that, alone with perceiving the awareness information from a brand, subjects didn’t need to allocate much attention resource to distinguish product with high brand awareness. The amplitude variation in N2 and P3 expressed the change in the identification and attention distribution processing. It can be concluded that EPR components N2 and P3 could serve as cognitive indexes to compare and measure the brand recognition and recall even as people didn’t pay special attention to the differences between brand awareness. Moreover, event-related potentials methodology may be a sensitive measurement technique for brand awareness.

## Data Availability Statement

All datasets generated for this study are included in the article/supplementary material.

## Ethics Statement

The studies involving human participants were reviewed and approved by the School of Management, Southwest University of Political Science and Law. The patients/participants provided their written informed consent to participate in this study.

## Author Contributions

The author confirms being the sole contributor of this work and has approved it for publication.

## Conflict of Interest

The author declares that the research was conducted in the absence of any commercial or financial relationships that could be construed as a potential conflict of interest.
